# Preliminary Characterization of “Salice Salentino” PDO Wines from Salento (South Italy) Negroamaro Grapes: NMR-Based Metabolomic and Biotoxicological Analyses

**DOI:** 10.3390/foods13223554

**Published:** 2024-11-07

**Authors:** Francesca Serio, Chiara Roberta Girelli, Mattia Acito, Giovanni Imbriani, Erika Sabella, Massimo Moretti, Francesco Paolo Fanizzi, Giuseppe Valacchi

**Affiliations:** 1 Department of Biological and Environmental Sciences and Technology, University of Salento, 73100 Lecce, Italy; francesca.serio@unisalento.it (F.S.); giovanni.imbriani@unisalento.it (G.I.); erika.sabella@unisalento.it (E.S.); fp.fanizzi@unisalento.it (F.P.F.); 2 Department of Pharmaceutical Sciences, University of Perugia, 06122 Perugia, Italy; mattia.acito@gmail.com (M.A.); massimo.moretti@unipg.it (M.M.); 3 Department of Environmental and Prevention Sciences, University of Ferrara, 44121 Ferrara, Italy; 4 Plants for Human Health Institute, North Carolina State University, Kannapolis, NC 28081, USA; 5 Department of Food and Nutrition, Kyung Hee University, Seoul 02447, Republic of Korea

**Keywords:** wine, protected designation of origin (PDO), bioactive compounds, metabolomics, ^1^H-NMR spectroscopy, human health, cytotoxicity, genotoxicity

## Abstract

(1) Background: A preliminary investigation of Protected Designation of Origin (PDO) wines (red and rosé) produced from Negroamaro grapes—a native Salento (Apulia, Southern Italy) vine that is part of the Salice s.no PDO area—was performed in this work. (2) Methods: ^1^H-NMR spectroscopy, in combination with multivariate statistical analysis (MVA), was employed to characterize the metabolic profiles of 39 wine samples. Spectrophotometric methods were used to obtain preliminary information on the phenolic composition of wines and the associated antioxidant activity. The HepG2 liver cell line was used to assess the biological activity (effect on cell viability and genotoxicity activity) of wine samples. (3) Results: The NMR spectra analysis revealed the presence of signals ascribable to phenolic compounds such as gallic, hydroxycinnamic, and syringic acids. Relative content of these metabolites has been shown to be higher in red than in rosés wines and related to the wine producers. Interestingly, a similar pattern was observed in biological analyses. Red wines compared to the rosé wines display great variations in antioxidant capacity when evaluated as fresh samples using the DPPH and ORAC methods. Furthermore, all red wines exhibited a concentration-dependent decrease in cellular viability and live cells; this phenomenon is much less pronounced in rosé wines. (4) Conclusions: The resulting findings from this study reveal that winemaking operations could lead to final products with different chemical compositions and related properties. Even when starting from the same crop variety and cultivation region, significant differences were observed in the wine samples NMR-metabolic profiles and in vitro biotoxicological activity.

## 1. Introduction

The qualities that customers want in agri-food products include hygienic and sanitary safety, hedonic and sensory quality, and nutritional value. Due to socioeconomic shifts brought about by the need to distribute goods over long distances and the increased competition from producers across borders, food products no longer always meet the high standards set by consumers, particularly when it comes to health. Indeed, consumers are becoming more conscious of the importance of healthy eating for maintaining health and preventing disease, including drinking high-quality wine to take advantage of its benefits. Wine is a beverage made from the alcoholic fermentation of the fruit of the *Vitis vinifera* L. (1753) vine. Its primary constituents are water (ca. 86%) and ethyl alcohol (ca. 12%), along with biologically active substances like tannins, polyphenols, organic acids, and vitamins that significantly influence the unique qualities of each wine [1,2]. Many studies have shown a correlation between moderate wine consumption and a decreased risk of developing chronic degenerative diseases, including cardiovascular diseases [3,4,5], cancer, diabetes, and hypertension [6]. While light to moderate drinking—typically one to two drinks per day in Western countries—has been linked to positive health impacts, ethanol intake also known as “drinking” has been linked to a number of detrimental consequences on health and quality of life [7,8]. According to Fernandes et al. [9], the benefits of wine are mostly determined by its chemical composition, which is directly related to the type of grape used, the terroir, the growth methods used, and the wine processing methods.

Due to its high polyphenol content, antioxidant properties in wine were already described [10]. As is known, polyphenols have been shown to have antioxidants properties mainly thanks to their ability to activate the cellular antioxidant defensive system Nrf2 [11]. Nevertheless, it should be highlighted that possible beneficial effects should be always linked to a moderate consumption of wine since excessive alcohol absorption can lead to increased health risks and mortality [12,13]. Moreover, wine can contain a variety of hazardous substances in addition to alcohol, resulting from manufacturing processes, environmental pollutants by human activity, improper handling, deceptive manipulation, or materials used in packaging [14,15]. Due to the presence of harmful compounds, researchers are interested in biomarkers of recent and past alcohol consumption to examine the dangers and potential advantages connected with it for nutritional assessment purposes [16] that have been already reported for red wine or beer [3,17,18,19]. Numerous studies [20,21,22,23,24] have been carried out to differentiate and/or authenticate the wine metabolome with the use of techniques like gas chromatography, mass spectrometry (GC-MS), and high-performance liquid chromatography (HPLC) [25,26]. These approaches have allowed us to classify wines according to characteristics like vintage, provenance, or technological processes [27,28]. Among scientific methods available in food sciences, metabolomics is widely used in food authenticity and traceability as well as geographical origin assessment studies [29,30].

Nuclear magnetic resonance (NMR) is one of most used analytical techniques in metabolomics, since it is a non-invasive, robust, and reliable quantitative approach that provides a snapshot of all the molecular components in a complex matrix. Although the wine chemical composition is complex since it is influenced by several factors (such as winemaking and storage conditions, agronomic practices, grape varieties), the ^1^H-NMR metabolomics approach has demonstrated to be a versatile tool for wine analysis and screening, providing meaningful information regarding the relationship between the product’s sensorial attributes and its primary metabolites [29,31,32,33]. Since it provides molecular scale-characterization of complex mixtures, the wine metabolic profiling could support the valorization, preservation, rediscovery, and cultivation of a native grape variety while opposing the marketing of inexpensive, low-fat wines that lack distinctive organoleptic qualities.

Although some chromatographic and NMR studies have been carried out on Apulian wines [32,34,35], few studies specifically focused on Negroamaro wine, an indigenous grape variety from Southern Italy. In 2014, De Pascali et al. [36] investigated a set of Negroamaro red wines obtained from different winemaking technologies and two soil management practices by ^1^H-NMR metabolomics and, more recently, Ragusa et al. [37] analyzed, using HPLC, the phenols in Negroamaro and Primitivo red wines from Salento. Thus, to the best of our knowledge, in this research we characterize, for the first time, the ^1^H-NMR-metabolic profiles and the biological properties of commercial red and rosé Negroamaro wine samples with a Protected Designation of Origin (PDO). This grapevine, which has code number 163 in the National Catalog of Vine Varieties [38], is grown in the provinces of Taranto, Brindisi, Foggia, Lecce, and Bari provinces, Apulia Region, Italy. Its preferred region is Salento, where it alone occupies more than 70% of the vineyard surface. This is because other environments lack critical elements necessary for this grape’s ideal maturation, such as the depth of the calcareous-clayey soils, which can act as a water reserve during protracted hot spells. Moreover, the ventilation and temperature variations, provided by the nearby seas, and extended exposure to heat and sunlight can promote sugars accumulation and allow the bunches to ripen sufficiently to counteract the significant number of acids and tannins. With its qualities determined by the Salento microclimates, the wine from this vine always has the same characteristics: a ruby red wine rich in coloring matter, a dry but fruity, velvety, and harmonious flavor, and a little bitter aftertaste. The phenolic fraction of the wine, in particular, has a significant influence on these sensory [39] as well as biological qualities like antioxidant, anti-inflammatory activity, and cardio- and cancer-protective properties [40,41]. Thus, a detailed Negroamaro metabolome characterization could highlight the presence of molecules with nutraceutical properties and, at the same time, possible differences among groups of wines [41]. By using this research methodology, information about the nutritionally and sensorially significant components of Negroamaro is obtained with the goal of enhancing our comprehension of its effects on human health or for supply chain process improvement. This adds value to its marketing and valorization for customers who are becoming more interested in a wine’s health benefits in addition to its flavor and heritage.

The aim of this study was to characterize by using a ^1^H-NMR-based chemometric analysis the “Salice Salentino” PDO wines’ metabolic profiles, identifying the presence of bioactive compounds and the potential differences related to the winemaking procedure. Moreover, the specific antioxidant activity, biotoxicological properties, and the related citotoxic/genotoxic activity were investigated by in vitro experiments. In this context, the present research could support the valorization, also for commercial purposes, of these value-added local products, with specific properties strictly related to the regions’ terroir and know-how [42].

## 2. Materials and Methods

**Figure 1:**
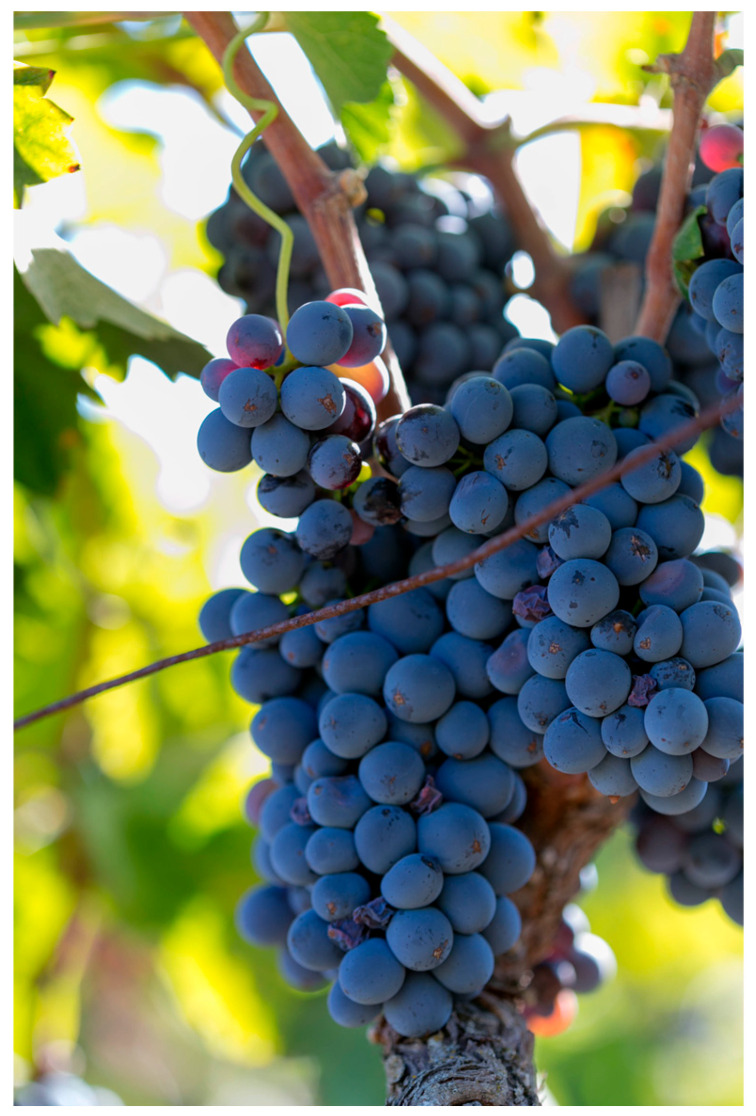
Negroamaro grapes on the vine in Apulia.

A total of 39 commercial bottles of red and rosé Negroamaro wines were supplied by seven trusted Salento wine producers (Table 1). All wine samples were certified by the Protected Designation of Origin (PDO), which certifies that a wine product only comes from grapes that are harvested in a particular region, thereby defining its quality standards and distinctive characteristics. In particular, all the analyzed samples here belong to the “Salice Salentino” Protected Designation of Origin (PDO). Specific production regulation [43] defines the conditions and requirements for the PDO labeling. The PDO designation is reserved for wines obtained from vinification of red and rosé grapes grown in vineyards which should be composed of at least 90% Negroamaro vine (Figure 1). To a maximum of 10% of the surface registered in the viticultural index, the grapes of other black grape varietals, suitable for growth in Apulia for the homogenous production region “Salento-Arco Ionico”, may also be used in the production of these wines, either alone or in combination.

The wine must be obtained from grapes grown in a well-defined geographical area that encompasses the administrative zone of Salice Salentino, Veglie, and Guagnano (Province of Lecce, Apulia, Italy), San Pancrazio Salentino and Sandonaci (Province of Brindisi, Apulia, Italy), and a part of the municipality of Campi Salentina and Cellino San Marco, Lecce and Brindisi Province, respectively. Environmental and cultivational conditions of vineyards for the PDO wines production must be those traditional to the area, or in any case, suitable to give grapes and wines their specific quality characteristics as specified in the PDO regulation [43]. The climate of this area is ideal for the cultivation of the vine: the mild winter and hot and dry summer, the strong thermal excursion, with the cool night breezes mitigating the heat of the day make this small peninsula the perfect place for the production of great wines. The maritime climate, with scarce and irregular rains and with sea breezes which mitigate the hot summer, makes the winter milder and shortens the spring and autumn seasons. The average temperature all year round is 16–17 °C, with maximum temperatures reaching 38–40 °C and a minimum of 2–7 °C. One of the Salento region’s famous rosé wines, which are among the world’s absolute finest, is bright coral pink in color, rich, opulent, and floral, with fine fruit on the nose. The first Italian rosé wine was bottled in the city of Salice Salentino, a record that shows the great value that the wine culture of this area gives to rosé winemaking. It is a rather difficult technique, requiring great skill to ensure that the same red wine grapes produce a delicate glass of rosé.

The information reported on the label of each wine commercial sample and the main physico–chemical parameters are reported in Table 1.

The winemaking operations of the PDO wine were covered by the rules established by the regulation [43]. In particular, it stated that the flesh of red wine grapes should be as transparent as white wine grapes. Its color should be obtained through the red color pigments in the skin of the red wine grapes. Unlike red wine production, where the grape skins are fermented together with the juice in a process known as “maceration”, in the rosé production, the skins are separated from the juice after just a few hours. The duration of the skin contact and the skin thickness determine the color of the final rosé [43]. The details of the winemaking processes, as declared by the different producers, are reported in Table 2.

### 2.1. Chemical Analyses

#### 2.1.1. NMR Chemicals and Reagents

All chemical reagents for analysis were of analytical grade. Deuterium oxide (99.9 atom %D) containing 0.05% wt 3-(trimethylsilyl)propionic-2,2,3,3 d4 acid sodium salt (TSP) potassium phosphate monobasic was purchased from Armar Chemicals (Döttingen, Switzerland). Sodium azide was purchased by J.T. Baker (Phillipsburg, NJ., USA).

#### 2.1.2. NMR Sample Preparation

Three technical replicates were obtained from each of the 39 commercial bottles of red and rosé “Salice Salentino” wines in order to minimize possible data inhomogeneity for a total of 117 samples. A volume of 900 µL of wines with 100 μL of a phosphate-buffered solution at pH 2.9 was added to each sample to reduce the pH variation range. The buffer contained 1 M KH_2_PO_4_ in D_2_O, 0.1% trimethylsilylpropanoic acid (TSP) as the internal reference standard and 2 mM NaN_3_ to prevent microbial contamination. The resultant solution was further centrifugated in order to eliminate possible solids (10,000× *g* at room temperature for 5 min). Then, a volume of 600 μL of the supernatant was transferred into an NMR tube (5 mm) for the NMR spectral acquisition and analysis [46].

#### 2.1.3. NMR Experiments

The NMR analyses were performed on a Bruker Avance III 600 Ascend NMR spectrometer (Bruker, Ettlingen, Germany) which operated at 600.13 MHz for ^1^H observation, equipped with a *z* axis gradient coil and automatic tuning-matching (ATM). All the NMR spectra were recorded at the temperature of 300 K by a Bruker Automatic Sample Changer, interfaced with the software IconNMR Version 5 (Bruker). A Bruker standard pulse program with presaturation for residual solvent signal (zgcppr) was used to acquire the NMR spectra with the following acquisition parameters: 128 scans (with 16 dummy scans) were collected at a time domain (TD) of 64 k data points, with a relaxation delay (RD) of 5.0 s. A spectral width (SW) of 12,019.230 Hz (20.0276 ppm) and an acquisition time (AQ) of 2.7262144 s were used. The resulting Free Induction Decay (FID) was multiplied by the exponential weighting function corresponding to a line broadening of 0.3 Hz before Fourier transformation, phase adjustment, and baseline correction. All spectra were referenced to the trimethyl silyl propionate standard (TSP) signal at 0.00 ppm. Spectra were processed using TopSpin 3.5 pl 7 (Bruker). Wine metabolites were identified and assigned on the basis of homo- and hetero-correlated 2D NMR experiments (2D ^1^H Jres, ^1^H-^1^H COSY, [^1^H,^13^C]-HSQC, and [^1^H,^13^C]-HMBC) and by comparison with previous works [33,46,47,48].

#### 2.1.4. NMR Data Processing and Statistical Analysis

The ^1^H NMR spectra were reduced into rectangular buckets (0.04 ppm width) and integrated by the Bruker Amix 3.9.15 (Analysis of Mixture, Bruker BioSpin GmbH, Rheinstetten, Germany) software. Binning procedure was performed within the 10.00–0.5 ppm range. The regions corresponding to the residual non-deuterated water (4.9–4.75 ppm) and ethanol (3.68–3.58 and 1.24–1.15 ppm) signals were eliminated and a total of 208 bins were further considered for statistical analysis. The NMR spectra were processed with TopSpin 3.5 pl 7 software (Bruker). With the aim to reduce the minor differences among samples, resulting from the metabolites concentration and/or experimental conditions, the total sum normalization was employed [49]. The Pareto scaling method was further used to the bucket reduced NMR spectra (variables). The data table resulting from all the aligned buckets row reduced spectra was used for multivariate data analysis. Each bucket in a buckets row reduced spectrum was labeled with the value of the central chemical shift for its specific 0.04 ppm width. The buckets are the variables used in chemometric analyses to perform multivariate statistical analysis. Unsupervised (principal component analysis, PCA) and supervised (orthogonal partial least squares discriminant analysis, OPLS-DA) pattern recognition methods were carried out using Software Simca-P version 14 (Sartorius Stedim Biotech, Umeå, Sweden). The PCA was applied to receive an overview of all observations in the data table by extracting the systematic variation in a data matrix X formed by rows (the studied observations) and columns (the variables) [50]. Hierarchical clustering analysis (HCA) was performed using the software “Metaboanalyst “version 5.0 [51] as a complementary data reduction and pattern recognition method on the data to measure the similarity among the groups. The resulting dendrogram was calculated using the Pearson distance algorithm and the heatmap developed by cluster aggregation was based on the Ward method [47,48,52]. OPLS-DA was further applied in order to highlight discriminating variables in the two class problems filtering out the variation not directly linked to the investigated discriminating response [50]. Validation of statistical models was performed by an internal cross-validation default method (7-fold) and the permutation test (100 permutations) [50]. The assessment of the models’ qualities was assessed by the R2X, R2Y, and Q2 parameter values, describing the total variation in X, the variation in the response variable Y, and the predictive ability of the models, respectively [53]. The statistical tool loadings plot was then used with the aim to highlight the variables responsible for the observed discrimination. The VIP and correlation coefficient p(corr) absolute values were employed to identify the potential discriminant metabolites [53]. Metabolites exhibiting variable importance in projection (VIP) and absolute p(corr) values larger than 1.0 and 0.5, respectively, were considered as potential discriminant metabolites [47,53]. The relative change in the content of the discriminating metabolite between the groups was estimated by calculating the mean values +/− standard deviation of selected bucket’s reduced NMR unbiased signal area after normalization to the total spectrum [47]. Student *t* test was used to assess the statistical significance of the differences between the means, assuming a *p*-value < 0.05 (confidence level 95%) as statistically significant.

### 2.2. Evaluation of Antioxidant Activity

#### 2.2.1. Determination of Total Polyphenolic Content (TPC)

A spectrophotometric Folin–Ciocalteu technique was used to estimate the total polyphenol content [54]. Wine samples were extracted using 80% ethanol (0.1 mL sample/10 mL of 80% ethanol); wine sample aliquots (0.2 mL) were added to 1.5 mL of newly made Folin–Ciocalteu reagent (1:10, *v*/*v*, with water) to calculate the total phenol contents (TPCs) of the extracts. After five minutes of equilibration, 1.5 mL of a 60 g/L sodium carbonate solution was added to the mixture. Following a 120 min incubation period at room temperature, the mixture’s absorbance was measured at 760 nm using the Victor X5–2030 Multilabel Reader (Perkin Helmer, Waltham, MA, USA) and the corresponding solvent as a blank. Gallic acid equivalents, or mg GAE, were used to express the results per liter of wine.

#### 2.2.2. Antioxidant Activity by DPPH (2,2-Difenil-1-picrylidrazyl) Assay

A total of 0.5 mL of each sample, properly diluted (1:10, 1:20, and 1:40), was combined with aliquots of 180 µL of DPPH (Sigma Aldrich, Taufkirchen, Germany) in methanol (9 mg/L) in a 96-well plate. Solution DPPH was employed as the reference sample. The absorbance was measured at 515 nm using the 96-well plate reader Victor X5–2030 Multilabel Reader (Perkin Helmer, Waltham, MA, USA) after 10 min of dark incubation at room temperature to track the absorbance drop. At least two experiments were performed, and all analyses on the examined samples were in triplicate. The following formula was used to compute the free radical scavenging activity, which was expressed as the percentage inhibition of DPPH:

Inhibition percentage (%) = [1 − (A10/A0)] × 100(1)

where A10 and A0 were values of absorbance of the test and blank samples, respectively. In order to determine the IC50 values, linear regression analysis was used. According to Sebaugh [55], the IC50 figure represents the concentration needed to achieve 50% inhibition with relation to the control.

#### 2.2.3. Antioxidant Activity by ORAC (Oxygen Radical Antioxidant Capacity) Assay

For the ORAC assay, the procedure was based on the method of Ou et al. [56]. The reaction was carried out in 75 mmol/L phosphate buffer (pH 7.4) and the final reaction mixture was 200 µL. The sample (20 µL) and fluorescein (120 µL; 70 nmol/L final concentration) solutions were placed in the well of the 96-well plate. The mixture was preincubated for 15 min at 37 °C beforehand. The 2,2′-Azobis(2-amidinopropane) di-hydrochloride (AAPH) solution (60 µL; 12 mmol/L final concentration) was added rapidly using a multichannel pipette. The plate was immediately placed in the reader (Victor X5–2030 Multilabel Reader, Perkin Helmer, Waltham, Massachusetts, USA) and the fluorescence recorded every minute up to value zero at 485 nm excitation, 520 nm emission. The plate was automatically agitated prior to each reading. All samples were analyzed in triplicate at three different dilutions. The final ORAC values were calculated using the differences in areas under the fluorescein decay curve between the blank and the sample and expressed in µmol Trolox equivalent (TE) per liter of analyzed wine.

### 2.3. Biotoxicological Analyses

Based on the preliminary NMR results, a set of 11 commercial bottles of PDO Salice Salentino Negroamaro red and rosé wine samples (i.e., eight red wine samples and three rosé wine samples) supplied by the selected seven trusted Salento winemakers were further investigated for their biological activity.

#### 2.3.1. Chemicals and Reagents

All reagents were analytical grade. Carlo Erba Reagenti Srl (Milan, Italy) provided the following supplies: ethanol, hydrochloric acid (HCl), ethylenediaminetetracetic acid disodium (Na_2_EDTA) and tetrasodium (Na_4_EDTA) salt, sodium chloride (NaCl), and sodium hydroxide (NaOH). Sigma-Aldrich Srl (Milan, Italy) provided the ethidium bromide, 4-nitroquinoline N-oxide (4NQO), tris (hydroxymethyl)aminomethane (Tris-HCl), low- and normal-melting-point agarose (LMPA and NMPA, respectively), and Triton X-100. Acridine orange (AO), NC-Slide A8TM and 6,4′-diamidino-2-phenylindole (DAPI) were procured from ChemoMetec A/S, located in Allerød, Denmark. Invitrogen Srl (Milan, Italy) supplied Eagle’s Minimum Essential Medium (MEM) and Dulbecco’s phosphate-buffered saline, pH 7.4 (PBS). Antibiotics (penicillin and streptomycin), fetal bovine serum (FBS), MEM non-essential amino acids (NEAA), sodium pyruvate, and trypsin were purchased from Euroclone SpA (Milan, Italy). Conventional microscope slides and coverslips were bought from Knittel-Glaser GmbH (Braunschweig, Germany).

#### 2.3.2. Extracts Preparation

After the chemical characterization, wine samples were tested for their biological activity. For each wine sample, two different extracts (Extract 1 and Extract 2) were prepared: one (Extract 1) resuspended in a solution of culture medium (MEM) and ethanol, the latter present in the same concentration (*v*/*v*, %) as present in the bottled product; the other one (Extract 2) resuspended exclusively in MEM. In particular, 90 mL of the product was divided into two sterile tubes (one tube for Extract 1 and one tube for Extract 2, 45 mL per tube) and freeze dried using a Christ^®^ ALPHA I/5 freeze dryer (Martin Christ Gefriertrocknungsanlagen GmbH, Osterode am Harz, Germania). After 24 h of lyophilization, the wines’ liquid component (water and ethanol) was completely removed. For each wine, Extract 1’s stock solution was prepared by resuspending the dry extract in 9 mL (5-fold concentration starting from the initial volume of 45 mL) of MEM and ethanol solution (the latter present in the same concentration as present in the bottled product), sonicated and vortexed until the extract was completely solubilized. Similarly, Extract 2’s stock solution was prepared by resuspending the dry extract with MEM, using an exact volume to obtain the same concentration of Extract 1’s stock solution (mg/mL). For each wine, Extracts 1 and 2 only differentiated for the presence, or not, of ethanol, allowing us to assess the potential interference of the latter on the activity of the biologically active molecules present in the extract. The concentration of each wine extract stock solution (mg/mL) is reported in Supplementary Table S1. Once prepared, the extracts were kept refrigerated (4 °C) until they were used in in vitro tests.

#### 2.3.3. Cell Line and Culture Conditions

Biological assays were carried out in HepG2 cells, a liver tumor cell line. Cells were provided by Istituto Zooprofilattico Sperimentale della Lombardia e dell’Emilia Romagna ‘Bruno Ubertini’ (Brescia, Italy). The HepG2 cells were cultured, as previously described [57,58], in 75 cm^2^ flasks in complete medium (MEM, 10% (*v*/*v*) FBS, 1% NEAA, 1 mM sodium pyruvate, 100 U/mL penicillin, and 0.1 mg/mL streptomycin) at 37 °C (humidified atmosphere, 5% CO_2_). Cells were subcultured by dispersal with 0.05% trypsin-EDTA solution (5 min) and plated (twice per week) at a 1:4 dilution. Experiments were performed on HepG2 cells at passages between 9 and 15.

#### 2.3.4. Cell Count and Viability: AO/DAPI Double Staining

The number of total and viable cells was assessed by staining cell populations with AO and DAPI fluorophores, as described previously [59,60]. Briefly, cells were plated in 12-well plates and maintained in culture for 24 h. After that, cells were treated with five scalar volumes (i.e., 36, 18, 9, 4.5, and 2.25 µL) of each extract stock solution or with 1% Triton X-100 (positive control) for four hours. In the case of EtOH-containing extracts (Extracts 1), the highest volume of stock solution used (i.e., 36 µL) contributed to a maximum EtOH concentration in the treatment which was always lower than 0.5% (*v*/*v*), which has been shown, in preliminary tests, to not affect cell viability in this cell line. Moreover, using this top stock solution volume, the top treatment concentration ranged, according to each extract, between 3.67 mg/mL (Wine 40) and 8.22 mg/mL (Wine 13) (Supplementary Table S1). This is also in accordance with the OECD (Organisation for Economic Co-operation and Development) Genetic Toxicology Guidance Document [61], which specifies that in cases when the test substance’s composition is unknown, the maximum dosage may exceed 2 mg/mL in order to raise the concentration of each component. Following the treatment, 38 µL of cell suspension and 2 µL of an AO/DAPI aqueous solution (AO: 30 µg/mL and DAPI: 100 µg/mL) were combined for each sample. Using NucleoCounter^®^ NC-3000TM (ChemoMetec A/S, Allerød, Denmark), a 10 µL aliquot of the resultant solution was put into the chambers NC-Slide A8TM and examined for cell count and viability.

#### 2.3.5. Genotoxicity Testing: COMET ASSAY

The comet assay was performed following a previously performed procedure, with minor modifications [62,63]. The detailed protocol is described elsewhere [59]. The air-dried slides were covered with a coverslip and stained with 50 µL of 20 µg/mL ethidium bromide right before scoring. Using an epi-fluorescent microscope (Nikon Eclipse E800, Nikon Instruments, New York, NY, USA) with a high-sensitivity black and white charge-coupled device (CCD) camera, the comets in each microgel were examined blindly at a magnification of ×200 (PE2020, Pulnix Europe Ltd., Basingstoke, UK) under a 100 W high-pressure mercury lamp (HSH-1030-L, Ushio Inc., Tokyo, Japan) using appropriate optical filters (excitation: 515–560 nm; emission: 590 nm). Comet Assay IV Lite Image Analysis Software (Perceptive Instruments, Suffolk, UK) was used to create microphotographs. Each sample consisted of two slides and fifty comets per slide. A total of one hundred randomly selected cells per sample were analyzed. The damage metric that was selected was tail intensity (%). To determine the group means, each slide’s scored comet median was employed [64,65].

#### 2.3.6. Statistical Analysis

The findings of the biological assays were summed up as the mean ± standard error of the mean (SEM) of three different experiments, following the Kolmogorov–Smirnov test to ensure that the data had a normal distribution. One-way analysis of variance (ANOVA) was used to compare the groups, and Dunnett’s post hoc analysis was used to compare each extract with untreated (control) cells. The differences between extracts that included and did not contain ethanol were ascertained for each wine using the paired sample *t*-test. For every statistical analysis, the threshold of significance was established at *p* < 0.05. The statistical analysis was conducted using SPSS (IBM Corp. Released 2010. IBM SPSS Statistics for Windows, Version 19.0. Armonk, NY, USA: IBM Corp.), a statistical software GraphPad Prism (GraphPad Software, Inc., San Diego, CA, USA) was used to create the graphs.

## 3. Results and Discussion

### 3.1. Chemical Analyses

#### 3.1.1. Metabolites Assignments in ^1^H-NMR Wine Sample Spectrum

**Figure 2:**
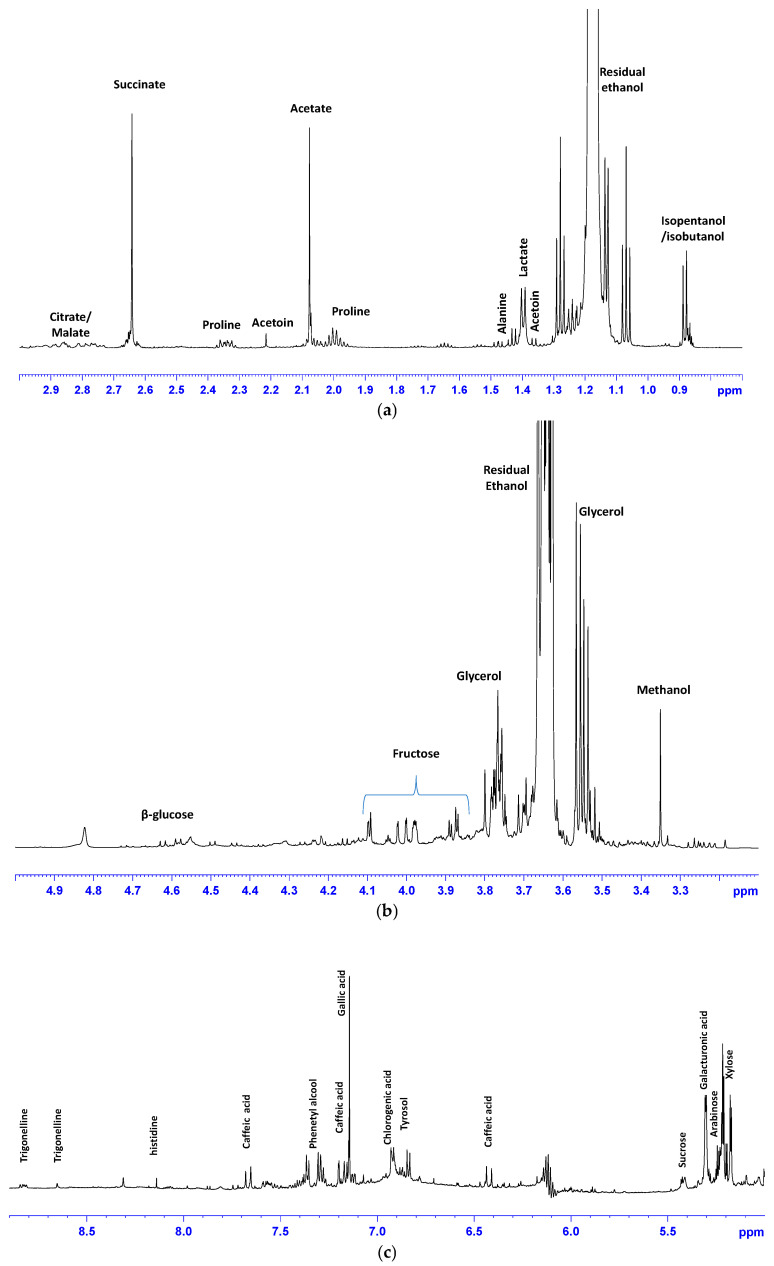
Representative ^1^H-NMR spectrum of a Negroamaro wine sample. Expansion of spectral regions in (**a**) 0.5–3 ppm aliphatic region; (**b**) (3–5 ppm) sugars region; (**c**) (5–10 ppm) aromatic region. The peaks of assigned metabolites are labeled.

Relative expansions of wine representative 600 MHz ^1^H-NMR spectral regions are shown in Figure 2a–c. As expected, the most intense peaks were ascribable to ethanol signals. In the low frequency field region of the spectrum (0.5–3.00 ppm) (Figure 2a), the presence of other alcohols (isobutanol, isopentanol), the aliphatic groups of amino acids (alanine, proline), and organic acids (lactic, acetic, succinic, citric, and malic) were detected. Moreover, signals of methyl ketone acetoin were identified. In the middle frequency region of the spectrum (range 3–5 ppm) high peaks assigned to glycerol were observed. Protons of β-glucose, fructose, methanol, and minor signals of myo-inositol and choline were also detected. The high frequency region of the spectra (5.0–9.5 ppm) showed signals assigned to sugars (α-glucose, sucrose, xylose, arabinose, galacturonic acid, and sucrose (Figure 2b). In the aromatic spectral region, proton resonances of catechin/epicatechin, chlorogenic acid, tyrosol, gallic acid, phenethyl alcohol, histidine, and trigonelline were observed (Figure 2c). A summary of the assigned metabolites is reported in Table 3.

#### 3.1.2. Multivariate Statistical Analysis

**Figure 3:**
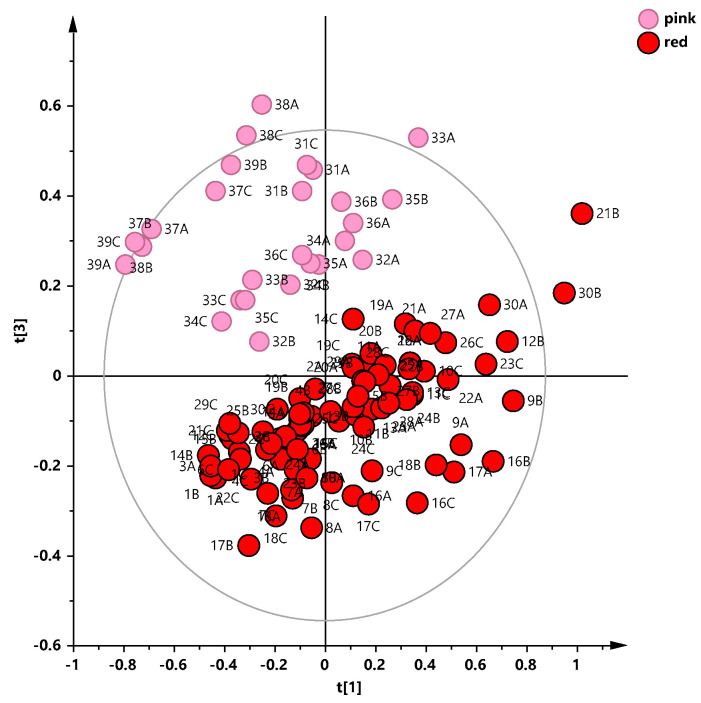
PCA t [1][3] scores plot (five components give R2X = 0.805, Q2 = 0.662) for whole wine sample data set. Sample symbols are colored according to different colors and labeled according to sample code.

The unsupervised PCA analysis was applied on the whole data set, after excluding two strong outliers, with the aim to obtain an overview of the data, identifying any possible grouping of the samples. The resulting model was made with five components and gave R2X = 0.805, Q2 = 0.662. An evident partition between red and rosé wine samples could be observed in the t1/t3 scores plot along the t [3] principal component (Figure 3). Interestingly, in the rosé wine samples group, a clear subclustering was identified, suggesting that the winemaking procedures could influence the wine metabolic profiles. In particular, the rosé wine was produced from the red grape skins whose duration contact and its thickness influenced the color of the final product [43]. Although further analyses are needed in order to confirm these preliminary observations, we could suppose that the skin-contact duration and/or the skin thickness for the different vineries (Table 1) could also influence the chemical features of the Salice Salentino rosé PDO wines.

**Figure 4:**
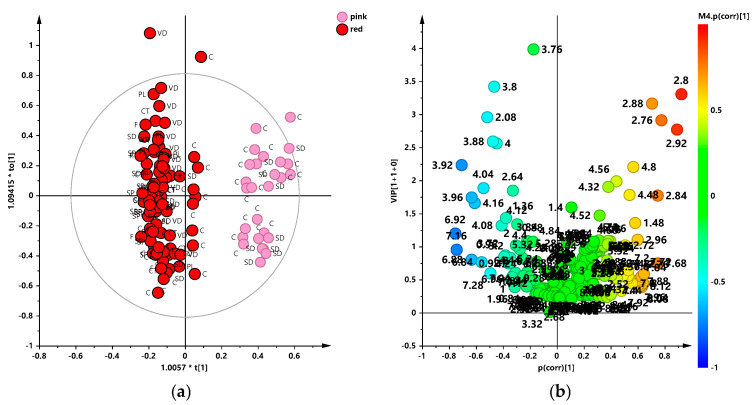
(**a**) OPLS-DA t [1]/t[2] scores plot for red and rosé wine samples classes (1 + 2 + 0; R2X = 0.614; R2Y = 0.917; Q2 = 0.912); (**b**) VIP plot for the model, colored according to the correlation scaled coefficient (p(corr), displayed for the first component). Symbols are colored according to different colors and labeled according to the different producers.

**Figure 5:**
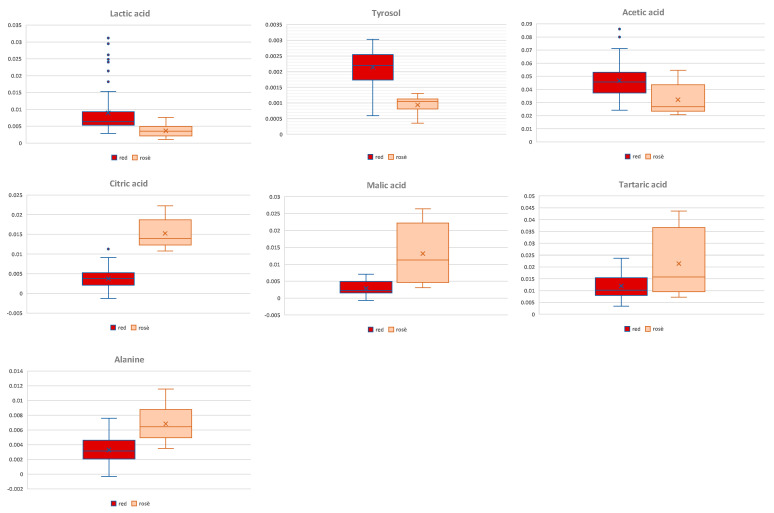
Box plots of significantly discriminant (*t*-test; *p* < 0.05) metabolites.

Thus, in order to obtain a deeper separation of the samples according to the different grape color, a supervised OPLS-DA was then performed considering the red and rosé color class samples. A good model was then obtained, with excellent descriptive and predictive parameters (1 + 2 + 0; R2X = 0.614; R2Y = 0.917; Q2 = 0.912), and revealed a strong separation between the two considered classes (Figure 4). The relative Volcano plot for the model showed the discriminating variables (NMR signals) according to the correlation scaled loading (p(corr)) and the variable importance in the projection (VIP) values. Quantitative comparison of the variables, characterized by the strongest contribution in the discrimination (|pcorr| ≥ 0.5) and strong discrimination power (VIP ≥ 1), was performed. This latter revealed that rosé wine exhibited a higher statistical (*p* value < 0.05) content of organic acids (malic, citric, and tartrate) and of amino acids (alanine) in comparison with the red ones which, on the contrary, showed a higher content of tyrosol, lactic, and acetic acids, as already described in the literature [34,66] and here reported for the first time for the Salice Salentino PDO wines (Figure 5).

**Figure 6:**
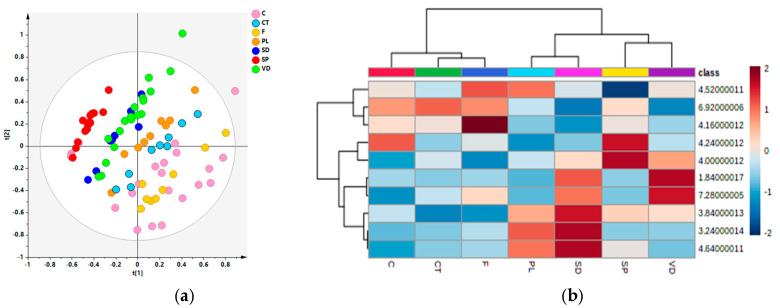
(**a**) PCA t[1]/t[2] scores plot for the whole red wine data set (5 components. R2(cum): 0.853; Q2(cum): 0.719). Sample symbols are colored according to the producers. (**b**) HCA dendrogram derived from ^1^H-NMR red wine samples, showing the clusters generated by a hierarchical cluster analysis of the data. The heatmap color indicates the normalized intensity of the bins: red, higher; gray, equal; blue, lower intensity.

Thus, with the aim to deeper analyze the wine metabolic profiles for the two considered classes, red and rosé samples were further investigated separately. The unsupervised (PCA) model (five components; R2X (cum): 0.853; Q2(cum) = 0.719), obtained for the red samples, showed an interesting distribution of the wines according to the producer. This is clearly detectable along a diagonal direction from negative and positive, respectively, to positive and negative values for the first two principal components of the PCA scores plot (Figure 6). In particular, visual inspection of the t[1]/t[2] scores plot revealed a quite clear separation specifically for the SP winery samples falling essentially at negative and positive values for the first two principal components t1/t2, respectively. On the other side, C and F winery samples were observed essentially at positive and negative values for the first two principal components t1/t2, respectively. Other samples constituted clusters located in intermediate positions, along the above-described diagonal direction, in the PCA scores plot. Thus, in order to remove any possible influence of the different wines harvest season, an unsupervised analysis was then applied on the wine samples from the same harvest year: 2018. Interestingly, the resulting PCA scores plot (Figure S1) showed similar trend grouping of the samples, confirming the effect of the winemaking operation on the metabolite composition. However, further research could be conducted in order to evaluate the influence of the different environmental factors (i.e., rainfall, seasonality, temperature) on the chemical composition of wines.

The HCA dendrograms showed descriptive results consistent with those of the PCA. Red wine samples, grouped on the basis of the producer, were spread across two different clusters (Figure 6b). Wine samples produced by C, CT and F belong to the same cluster. This grouping is mainly characterized by lower intensity of β-glucose (bins at 3.24, 3.84, 4.64), fructose (bin at 4), phenethyl alcohol (bin at 7.28), and proline (bin at 1.84). In the second cluster, red wine samples from PL, SD, SP, and VD producers were split into two main subclustering characterized by the higher (PL and SD) and lower (SP and VD) intensity of β glucose-assigned bins (3.24, 3.84, 4.64).

**Figure 7:**
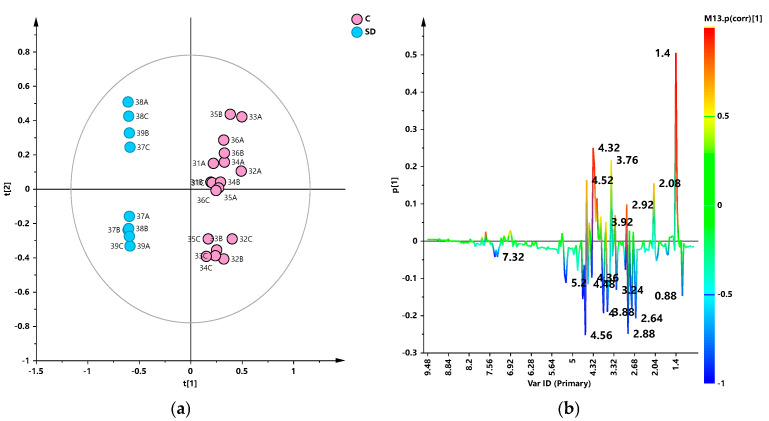
(**a**) PCA t[1]/t[2] scores plot for the whole rosé wine data set (4 components. R2X(cum) = 0.94; Q2(cum) = 0.878). Sample symbols are colored according to the producers and labeled according to the sample code. (**b**) Loading line plot for the model colored according to the correlation scaled coefficient p(corr), displayed for the first component.

In the case of PCA performed on the rosé samples, a clear partition of the samples along the first principal component according to the producers was observed (Figure 7a). The t[1]/t[2] scores plot for the model, built with four components (R2X(cum) = 0.94; Q2(cum) = 0.878) clearly showed the separation between the two wine sample producers, also revealing a clear intra-group variability along the second component, not ascribable to the different wine vintage (see Table 1). The loading line for the model (Figure 7b) showed the metabolite responsible for the discrimination: relative content of acetoin (bin at 1.4), acetic acid (2.08), citrate (2.9), and tartrate (4.52) was higher in C producer and, on the contrary, isobutanol (0.88), malic acid (2.64, 2.88), α-glucose (5.2), fructose (4), and phenethyl alcohol (7.32) were found discriminating for the SD producer. We could hypothesize that the observed differences in the metabolic profiles of wine samples from different producers could be related to differences in the vinification procedures such as the skin-contact duration (Table 1). Moreover, in this case, in order to check the possible effect of the seasonality on the wine metabolic profiles, a PCA was conducted on the rosé wine samples from the same harvesting year. As described for the red samples, visual inspection of the PCA scores plot (Figure S2) showed a nice clear partition of the samples according to the different wineries.

### 3.2. Biological Analyses

#### 3.2.1. Total Polyphenolic Content

The Folin–Ciocalteu assay is a helpful test to obtain initial information about the total phenolic content of a material, despite its lack of specificity due to potential interference from sugars, ascorbic acid, aromatic amines, and other compounds. Total phenolic content in wine samples varied from 571.3 ± 3.3 to 1463.2 ± 9.2 mg GAE/L wine (Table 3) for rosé and red wines, respectively, showing that the samples of red wines had more phenolic content than the samples of rosé wines. These data are in accordance with the current literature that has evidenced that red wines have a significantly higher phenolic content and antioxidant capacity than rosé wines [65]. Specifically, Banc et al. [67] studied the phenolic profile and composition in relation to the antioxidant activity of fifteen samples of commercial red and rosé wines from six native grape varieties produced in Romania. The concentration ranges that were obtained agreed with the values stated in the literature [68,69,70,71,72]. Fermo et al.’s [73] investigation concentrated on the polyphenol content of Italian wines from three distinct locations, each with a unique provenance and kind. Among the wines under analysis, Grechetto displayed a distinctive polyphenol profile with higher levels of p-coumaric acid, caffeic acid, and catechin, as well as gallocatechin, which was unique to this kind of wine. Total polyphenol content (TPC) values and metabolite profiles strongly depend on grape genotype. In any case, when the TPC was compared to the other rosé and red wines, only wine numbers 32 and 39 showed better values (Table 4).

#### 3.2.2. Antioxidant Activity of Wine Samples

**Figure 8:**
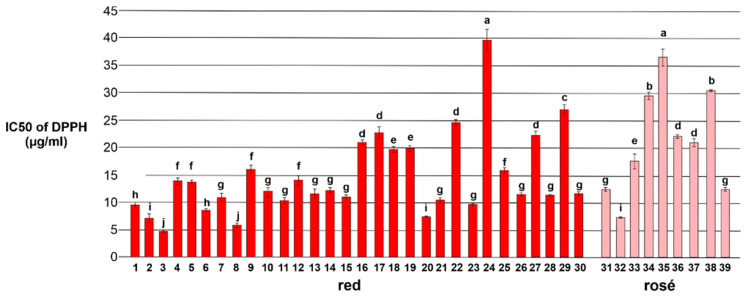
IC50 values for DPPH-free radical scavenging activity of the red and rosé wines from Negroamaro grapes. Different letters indicate statistically different means (Duncan’s test, *n* = 3, *p* < 0.05).

The lowest IC50 value indicates the highest antioxidant activity of the analyzed wines. The antioxidant activity values ranged between 4.60 ± 0.70 and 39.60 ± 1.32 µg/mL in red wines (Figure 8). Red wines, in general, display great variations in antioxidant capacity when evaluated as fresh samples via the DPPH method [74]. However, compared to the rosé wines and to findings published elsewhere [75,76], our red wines ranked relatively high. Although not specific, spectrophotometric methods were useful to obtain preliminary information of the phenolic composition of wines and the associated antioxidant activity.

The ORAC assay was used as an additional method to evaluate antioxidant activities of the analyzed wines and the results are presented in Table 5. The antioxidant activity values ranged between 8122 ± 161 and 16,246 ± 178 µmol Trolox equivalent (TE) L^−1^ in red wines and 8421 ± 163 and 12,791 ± 193 µmol Trolox equivalent (TE) L^−1^ in rosé wines.

Total phenolic contents (Table 4) and the antioxidant activity (Figure 8 and Table 5) measured with the DPPH and the ORAC assays were significantly correlated (respectively, r = −0.86, *p*-value = 4.17 × 10^−9^, and r = 0.73, *p*-value = 1.153 × 10^−5^) according to the Pearson’s correlation test. The two antioxidant assays versus the total phenolic content showed a similar correlation coefficient; thus, the Pearson correlation coefficient measured for the DPPH test showed a negative value as expected due to the opposite representation of the result (of the IC50. The high correlation obtained supports the fact that antioxidant activity is mainly due to the phenolic compounds.

Many antioxidants, including phenolic acids catechin, epicatechin, rutin, quercetin, myricetin, and anthocyanins, procyanidins, and pyroanthocyanidins, as well as others, have been suggested to be the cause of red wine’s demonstrated antioxidant capabilities [77,78]. Although the ability of these compounds to quench ROS has been shown in vitro, their main antioxidant role is related to the activation of the antioxidant master regulator Nuclear Factor (Erythroid-Derived 2)-Like 2 (Nrf2) which is a transcription factor that activates a battery of cytoprotective genes mainly involved in preventing oxidative damage and inflammation [79].

Additionally, research has shown that red wines include more polyphenols than rosé wines and that these polyphenols contribute to stronger antioxidant activity [44,62,80]. The polyphenol content and contribution of thirteen distinct Italian wines to the total antioxidant capacity were studied by Simonetti et al. [81]. Red wines have a high level of flavonols (average 15.3 mg/L), with the most prevalent being rutin and quercetin, followed by myricetin, kaempferol, and isorhamnetin, which together accounted for just 0.7−3% of total antioxidant activity (TAA). Many other studies all around the world have conducted studies on the antioxidant activity of red wine confirming the above-mentioned literature [82,83,84,85,86,87,88,89,90,91].

### 3.3. Biotoxicological Analyses

#### 3.3.1. Cell Count and Viability

**Figure 9:**
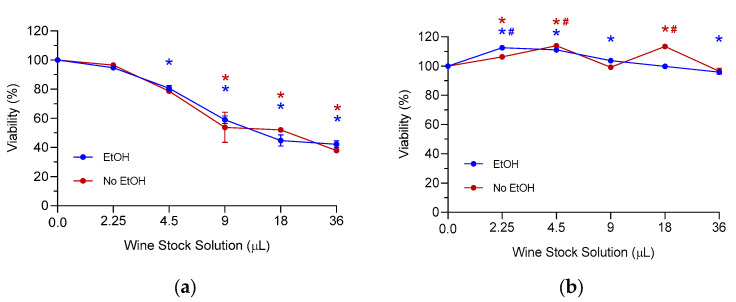
Percentages of viable HepG2 cells after treatment with Wine Stock Solution. (**a**) Representative red wine from Negroamaro grapes (sample *n*. 10); (**b**) representative rosé wine from Negroamaro grapes (sample *n*. 37). The results are summarized as three independent experiments’ mean (±SEM). Results are reported as variations with respect to the untreated control (taken as 100%). Statistical analysis: one-way ANOVA followed by Dunnett’s post hoc for treated samples vs. control (untreated) cells (* *p* < 0.05, treated vs. control); paired sample *t*-test was used to determine the difference between EtOH-containing and EtOH-free extracts (# *p* < 0.05, EtOH-containing vs. EtOH-free).

The representative effect of wine extracts on HepG2 viability is illustrated in Figure 9. The whole pattern of samples is shown in Figure S3. Concerning cell viability, our results highlighted the differences between the types of wine studies. Independently of the presence of ethanol:

(i) All red wine samples were able to remarkably affect cell viability in a concentration-dependent manner;
(ii) All rosé wine samples showed mixed trends, with little impact on cell viability.

#### 3.3.2. Genotoxicity Testing

**Figure 10:**
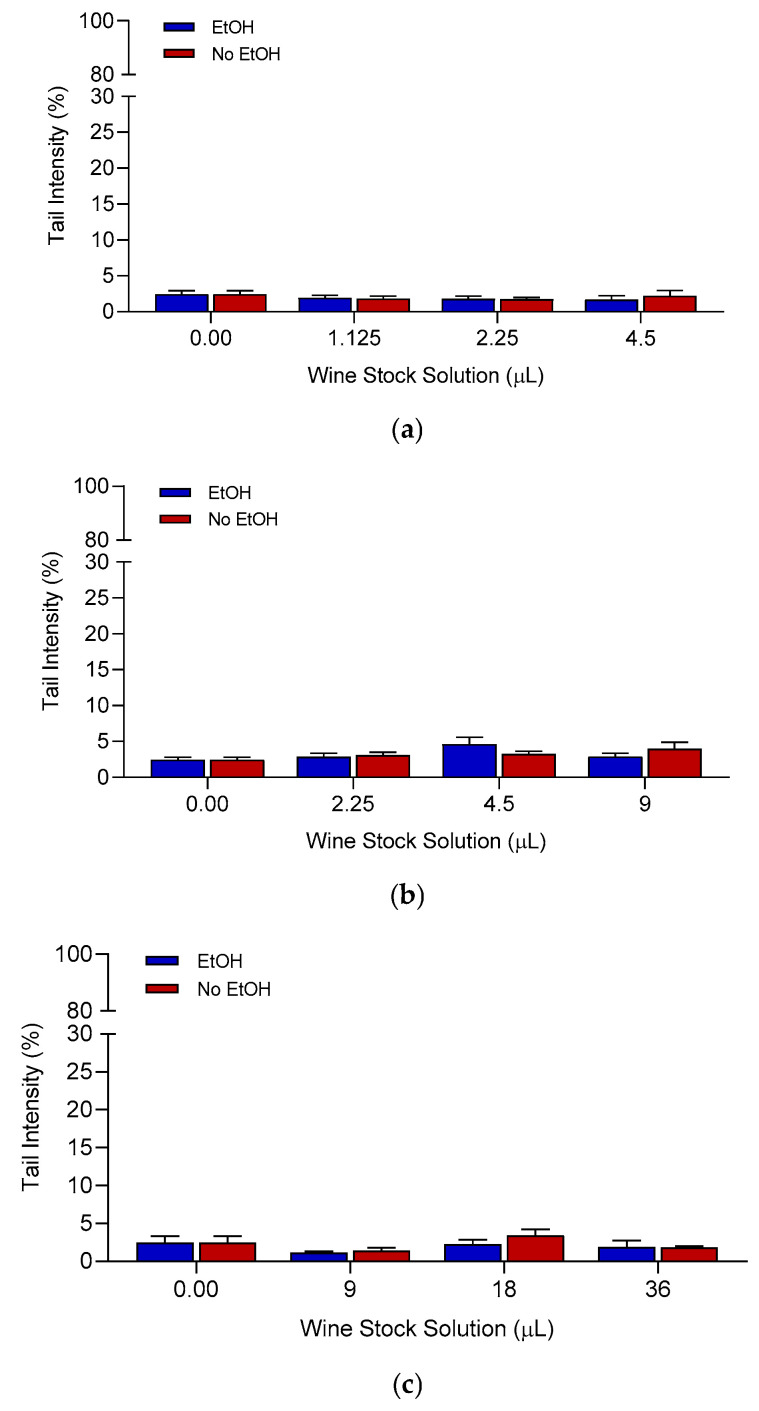
Tail intensity (% DNA) was used as an indicator of primary DNA damage. (**a**) Representative red wine from Negroamaro grapes (sample *n*. 10); (**b**) representative red wine from Negroamaro grapes (sample *n*. 25); (**c**) representative rosé wine from Negroamaro grapes (sample *n*. 37). The results are summarized as three independent experiments’ mean (±SEM). Statistical analysis: one-way ANOVA followed by Dunnett’s post hoc for treated samples vs. control (untreated) cells; paired sample *t*-test was used to determine the difference between EtOH-containing and EtOH-free extracts. Tail intensity of positive control (1 µM 4NQO): 12.31 ± 1.15 (*p* < 0.001 vs. untreated cells, *t*-test).

For each wine sample, genotoxicity testing (comet assay) was performed using the three highest concentrations that did not show cytotoxic effects in the AO/DAPI assay. Representative examples of the genotoxic effect of the wine extracts on HepG2 cells are illustrated in Figure 10. The whole pattern of samples is shown in Figure S4. Except for some sporadic significant increases in the tail intensity values in some of the samples, overall, wine samples were unable to induce primary DNA damage in HepG2 cells. Also, similar to the viability assay, in most cases there were no statistically significant differences between EtOH-containing and EtOH-free samples, with similar trends.

## 4. Conclusions

A ^1^H-NMR based metabolomic method and biotoxicological analyses were utilized to characterize, for the first time, the PDO Salice Salentino red and rosé wines chemical compositions. Our findings revealed the presence of compounds with bioactive properties such as gallic, hydroxycinnamic, and syringic acids. Interestingly, as well as the expected differences in the red and rosé wines metabolic profiles, as also confirmed by the antioxidant activity comparison, meaningful differences among wines from different producers were observed. This preliminary investigation suggested that the producer-specific winemaking procedure, although within the limits established by the specific PDO regulation, could influence the metabolic profiles of the wines. Although further analysis would be useful to deeply characterize the chemical and biological features of PDO Negroamaro wines, also taking into account the possible effect of the harvest season and other environmental factors as temperature and rainfall, on the metabolite composition, the NMR-based metabolomics protocol used here could be proficiently used, by the producers, as a tool for the traceability assessment of their products. Moreover, the biological assays showed that, although from the same grape variety—Negroamaro—whose cultivation is allowed only in a very limited area, the different vinification methods could also produce distinct features that go well beyond the organoleptic viewpoint. When compared with rosé wines, red wine samples were significantly more “active” toward HepG2 cancer cell lines. It is possible to hypothesize that the observed biological effects could be mainly related to the variable presence of bioactive phytochemicals in each sample, given that we saw superimposable trends within the majority of the samples between EtOH-containing and EtOH-free samples. Additional analyses would be advisable in order to deeply investigate the biological properties, opening the door for additional primary cell research.

## Figures and Tables

**Table 1:** List of analyzed “Salice Salentino” PDO wine samples.

Winery *	Number of Bottles	Color	Variety	Alcohol Percentage (%) **	Total Acidity (g/L) **	Sugars (g/L) **	pH **	Sulfur Dioxide (mg/L) **	Harvesting Period
SP	6	red	100% Negroamaro	13.5	5.4 ± 0.28	1.68 ± 0.16	3.3 ± 0.1	107.0 ± 0.4	2018
F	3	red	100% Negroamaro	13	6.4 ± 0.16	0.98 ± 0.09	3.4 ± 0.01	89.0 ± 0.8	2016
PL	3	red	85% Negroamaro 15% other cultivars permitted in the production regulation [43]	13.5	5.8 ± 0.2	0.66 ± 0.09	3.45 ± 0.02	94.4 ± 2	2018
SD	3	red	100% Negroamaro	13.5	5.69 ± 0.06	1.48 ± 0.16	3.3 ± 0.00	96 ± 2	2018
C	3	red	100% Negroamaro	14	5.89 ± 0.06	0.6 ± 0.09	3.45 ± 0.01	94.7 ± 0.5	2019
C	3	red	100% Negroamaro	14	5.66 ± 0.2	1.53 ± 0.15	3.2 ± 0.02	95.8 ± 3	2018
CT	3	red	95% Negroamaro 5% Malvasia	14	6.2 ± 0.23	1.54 ± 0.16	3.4 ± 0.00	90 ± 0.7	2018
VD	6	red	80% Negroamaro 20% Malvasia	14	5.8 ± 0.2	1.20 ± 0.12	3.0 ± 0.02	92.0 ± 0.2	2017
C	3	rosé	95% Negroamaro 5% Malvasia	13.5	5.5 ± 0.21	0.1 ± 0.05	3.5 ± 0.03	87.6 ± 0.8	2019
C	3	rosé	95% Negroamaro 5% Malvasia	13.5	5.9 ± 0.2	0.8 ± 0.07	3.4 ± 0.01	99.0 ± 2	2018
SD	3	rosé	95% Negroamaro 5% Malvasia	13.5	6.1 ± 0.2	0.9 ± 0.03	3.3 ± 0.00	85.0 ± 1	2019

* SP, F, PL, SD, C, CT, and VD codes indicates the different wineries. ** The physico–chemical parameters: residual sugar, sulfur dioxide content, minimal alcohol level, pH, and total acidity of the wines were assessed by analytical methods according to the International Organisation of Vine and Wine (OIV) [44] The obtained values were in accordance with the limits established by the Consolidated Wine Act [45] and the PDO production rules [43].

**Table 2:** List of the winemaking procedures for the different wineries.

Winerie	Declared Winemaking Procedure
Red wines	
SP	Soft destemming and pressing, temperature (26 °C). Controlled fermentation in heat-conditioned stainless-steel tanks for 8–10 days. Aging in stainless-steel for 6 months.
F	Fermentation at a controlled temperature for 8 days; short post-fermentative maceration. Spontaneous malolactic fermentation at the end of alcoholic fermentation. Aging in stainless steel tanks.
PL	Soft destemming and pressing, temperature-controlled (25 °C). Fermentation in heat-conditioned stainless-steel tanks for 12 days. Aging in stainless steel tanks.
SD	Soft destemming and pressing, temperature-controlled (25 °C). Fermentation in heat-conditioned stainless-steel tanks for 12 days. Aging in stainless steel tanks.
C	After maceration, selected yeasts were added and fermentation took place under rigorous temperature control. Aging in small casks for 8 months.
CT	De-stemming and pressing, follow soft pressing; temperature-controlled fermentation (25 °C) in heat-conditioned stainless-steel tanks for a period of approximately 18–20 days. Aging in stainless-steel for 6 months.
VD	After de-stemming and pressing red vinification in steel tanks at a controlled temperature between 22° C and 28° C. Aging in stainless-steel for 12 months.
Rosé wines	
C	The destemmed and crushed berries were cold macerated for some 20 h after which a part of the juice (less than 40%) was drained off and fermented in stainless steel using cultured yeasts. Aging in stainless steel tanks.
SD	According to the tradition, after the fermentation on the skins for 10–12 h the must was drained off obtaining about 40% of the total product. Aging in stainless steel tanks.

**Table 3:** ^1^H-NMR chemical shifts in assigned wine metabolites.

Compounds	Chemical Shifts (δH)
Isobutanol	0.88
Isopentanol	0.89; 1.64
Ethanol	1.18; 3.65
Acetoin	1.4
Lactic acid	1.43
Alanine	1.47
Proline	2.00; 2.35
Acetic acid	2.08
Succinic acid	2.65
Malic acid	2.76; 2.87; 4.48
Citric acid	2.79; 2.92
Choline	3.18
Myo-inositol	3.26
Methanol	3.34
Glycerol	3.55; 3.64; 3.77
Fructose	3.88; 4.00; 4.10
Arabinose	4.50
Tartrate	4.53
β-glucose	4.65
Xylose	5.17
α-glucose	5.22
Arabinose	5.25
Galacturonic acid	5.30
Epicatechin	6.12
Chlorogenic acid	6.43; 7.14; 7.21; 7.68
Fumaric acid	6.70
Tyrosol	6.84; 7.18
Gallic acid	7.15
Phenethyl alcohol	7.31; 7.37
Histidine	8.14
Trigonelline	9.18; 8.93; 8.09

**Table 4:** Total phenolic content (TPC), expressed as mg of gallic acid equivalents (GAE) per liter of the analyzed wine. Different letters correspond to statistically different means (Duncan’s test, *n* = 3, *p* < 0.05).

Wine Samples	Total Phenolic Content (mg GAE/L Wine)
1	1325.2 ± 2.3 ^b^
2	1225.1 ± 4.4 ^d^
3	1463.2 ± 9.2 ^a^
4	912.3 ± 2.9 ^h^
5	975.4 ± 3.4 ^g^
6	1289.9 ± 2.3 ^c^
7	1142.6 ± 1.9 ^e^
8	1355.4 ± 7.1 ^b^
9	931.9 ± 2.8 ^h^
10	1015.7 ± 2.4 ^g^
11	1047.3 ± 3.6 ^f^
12	984.4 ± 3.1 ^g^
13	1124.5 ± 2.2 ^e^
14	1263.3 ± 2.9 ^c^
15	1003.7 ± 3.4 ^g^
16	751.9 ± 3.1 ^i^
17	758.1 ± 3.9 ^i^
18	746.8 ± 3.3 ^i^
19	1227.9 ± 2.8 ^d^
20	1221.8 ± 3.5 ^d^
21	1236.3 ± 3.4 ^d^
22	578.7 ± 2.4 ^j^
23	991.5 ± 2.6 ^g^
24	745.8 ± 2.7 ^i^
25	954.8 ± 2.1 ^g^
26	1145.2 ± 2.9 ^e^
27	696.8 ± 3.4 ^i^
28	982.7 ± 2.2 ^g^
29	761.2 ± 3.3 ^i^
30	1199.1 ± 3.4 ^d^
31	1013.9 ± 2.9 ^g^
32	1136.1 ± 3.2 ^e^
33	893.6 ± 4.1 ^h^
34	743.2 ± 2.5 ^i^
35	571.3 ± 3.3 ^j^
36	895 ± 2.8 ^h^
37	883.7 ± 1.8 ^h^
38	622.3 ± 3.1 ^j^
39	1147.3 ± 2.1 ^e^

**Table 5:** Antioxidant activity of red and rosé wines determined by the ORAC method; the data are expressed as µmol Trolox equivalent (TE) per liter of analyzed wine. Different letters correspond to statistically different means (Duncan’s test, *n* = 3, *p* < 0.05).

Wine Samples	ORAC (µmol TE/L) ^b^
1	15,750 ± 165 ^b^
3	16,246 ± 178 ^a^
4	11,336 ± 198 ^f^
5	11,265 ± 101 ^f^
6	13,822 ± 191 ^c^
7	13,002 ± 199 ^d^
8	14,204 ± 201 ^c^
9	11,393 ± 187 ^f^
10	12,022 ± 185 ^e^
11	12,114 ± 194 ^e^
12	11,612 ± 188 ^f^
13	12,895 ± 192 ^d^
14	13,217 ± 199 ^d^
15	11,983 ± 103 ^e^
16	9862 ± 168 ^h^
17	9494 ± 173 ^h^
18	9451 ± 166 ^h^
19	12,954 ± 190 ^d^
20	13,222 ± 193 ^d^
21	13,164 ± 101 ^d^
22	8122 ± 161 ^j^
23	11,012 ± 183 ^f^
24	9398 ± 173 ^h^
25	11,125 ± 191 ^f^
26	12,998 ± 196 ^d^
27	9015 ± 168 ^i^
28	11,215 ± 184 ^f^
29	9443 ± 181 ^h^
30	12,236 ± 194 ^e^
31	12,128 ± 187 ^e^
32	11,994 ± 193 ^e^
33	10,322 ± 182 ^g^
34	9374 ± 178 ^h^
35	8421 ± 163 ^j^
36	10,427 ± 188 ^g^
37	10,121 ± 192 ^g^
38	9333 ± 166 ^h^
39	12,791 ± 193 ^d^

## Data Availability

Data are contained within the article and in the Supplementary Materials.

## Supplementary Materials

The following supporting information can be downloaded at: https://www.mdpi.com/article/10.3390/foods13223554/s1, Table S1: Information concerning stock solution concentration, highest extract and EtOH concentrations for each wine extract. Figure S1: PCA t[1]/t[2] scores plot for the red wine data set from the 2018 harvest year (5 components. R2(cum): 0.933; Q2(cum): 0.845). Figure S2: PCA t[1]/t[2] scores plot for the rosé wine data set from the 2019 harvest year (5 components. R2(cum): 0.965; Q2(cum): 0.89). Sample symbols are colored according to the producers. Figure S3: Percentages of viable HepG2 cells after treatment with Wine Stock Solution. Figure S4: Primary DNA damage in HepG2 cells after treatment with Wine Stock Solution.
