# Supplementary material for: Preliminary Characterization of “Salice Salentino” PDO Wines from Salento (South Italy) Negroamaro Grapes: NMR-Based Metabolomic and Biotoxicological Analyses
Source: Foods. 2024 Nov 7;13(22):3554. doi: 10.3390/foods13223554 (PMC11592925; doi:10.3390/foods13223554)
Supplement: Supplementary file 1 [file foods-13-03554-s001.zip › Serio_Supplementary_Material.pdf]

Supplementary Materials

Preliminary characterization of "Salice salentino" PDO wines from Salento (South Italy) Negroamaro grapes: NMR-based metabolomic and biotoxicological analyses

Francesca Serio[1], Chiara Roberta Girelli[1*], Mattia Acito[2], Giovanni Imbriani[1], Erika Sabella[1], Massimo Moretti[2], Francesco Paolo Fanizzi[1], Giuseppe Valacchi[3,4,5]

[1] Department of Biological and Environmental Sciences and Technology, University of Salento, 73100 Lecce, Italy; francesca.serio@unisalento.it (F.S.); giovanni.imbriani@unisalento.it (G.I.); erika.sabella@unisalento.it (E.S.); fp.fanizzi@unisalento.it (F.P.F.);
[2] Department of Pharmaceutical Sciences, University of Perugia, 06122 Perugia, Italy; mattia.acito@gmail.com (M.A.); massimo.moretti@unipg.it (M.M.);
[3] Department of Environmental and Prevention Sciences, University of Ferrara, 44121 Ferrara, Italy; vlcgpp@unife.it (G.V.);
[4] Plants for Human Health Institute, North Carolina State University, Kannapolis 28081, NC, USA; gvalacc@ncsu.edu (G.V.);
[5] Department of Food and Nutrition, Kyung Hee University, Seoul 02447, South Korea, (G.V.);
* Correspondence: chiara.girelli@unisalento.it (C.R.G.) Tel.: +39-0832-298715;

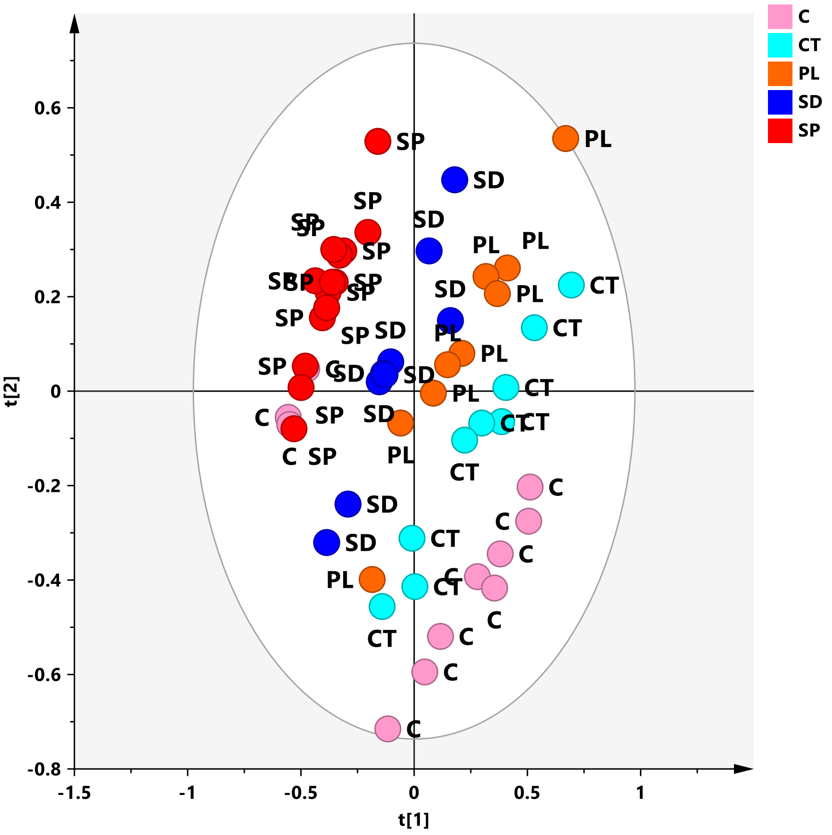

Figure S1. PCA t[1]/t[2] scores plot for the red wine data set from 2018 harvest year (5 components. R2(cum): 0.933; Q2(cum): 0.845). Sample symbols are coloured according to the producers

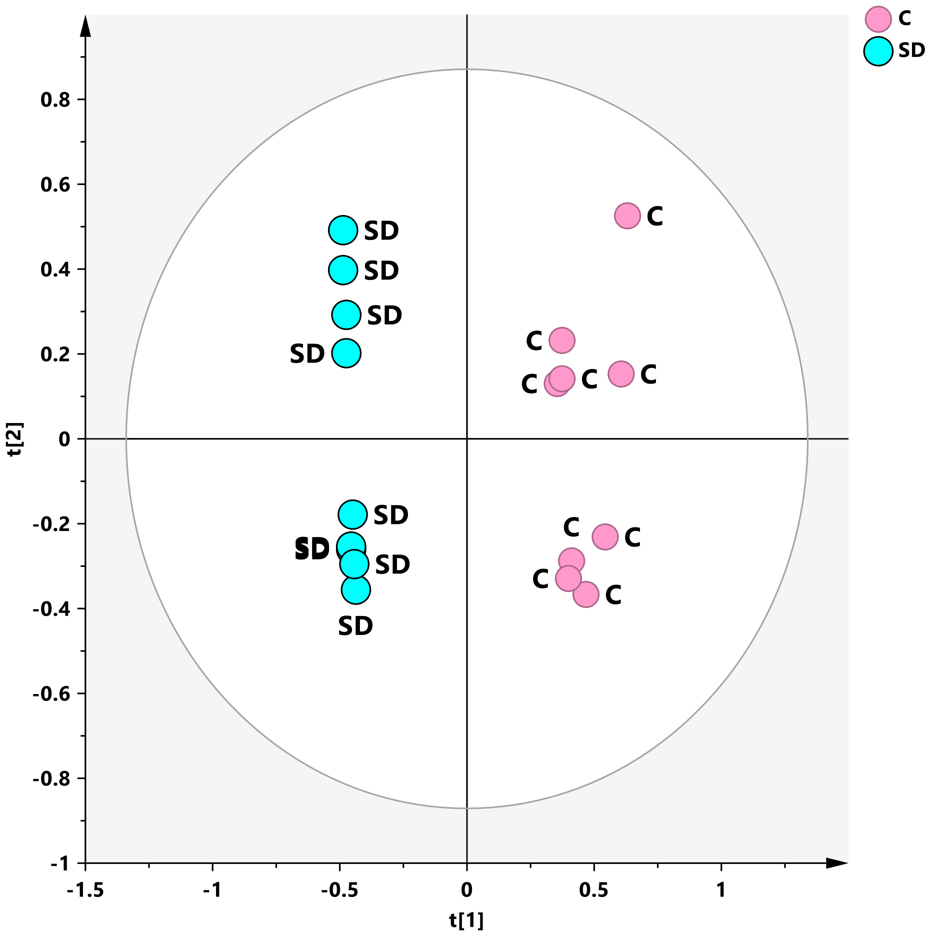

Figure S2. PCA t[1]/t[2] scores plot for the rosè wine data set from 2019 harvest year (5 components. R2(cum): 0.965; Q2(cum): 0.89). Sample symbols are coloured according to the producers

Supplementary Table S1. Information concerning stock solution concentration, highest extract and EtOH concentrations for each wine extract.

Wine Extract	EtOH content in wine (%)	Stock solution concentration (mg/mL)	Highest extract concentration in treatment [a] (mg/mL)	Highest EtOH concentration in treatment [b] (%)
**1**	13.5	217.14	7.82	0.48
**7**	13	154.82	5.57	0.46
**10**	13.5	195.21	7.03	0.48
**13**	13.5	228.27	8.22	0.48
**16**	14	160.57	5.78	0.50
**19**	14	177.31	6.38	0.50
**22**	14	169.71	6.11	0.50
**25**	14	196.31	7.07	0.50
**31**	13.5	110.82	3.99	0.48
**34**	13.5	150.40	5.41	0.48

**37**	13.5	113.95	4.10	0.48

[a] resulting from 0.036 mL of stock solution in 1 mL of total treatment volume

[b] this information exclusively refers to EtOH-containing extracts (Extract 1 of each wine sample)

Figure S3. Percentages of viable HepG2 cells after treatment with Wine Stock Solution.

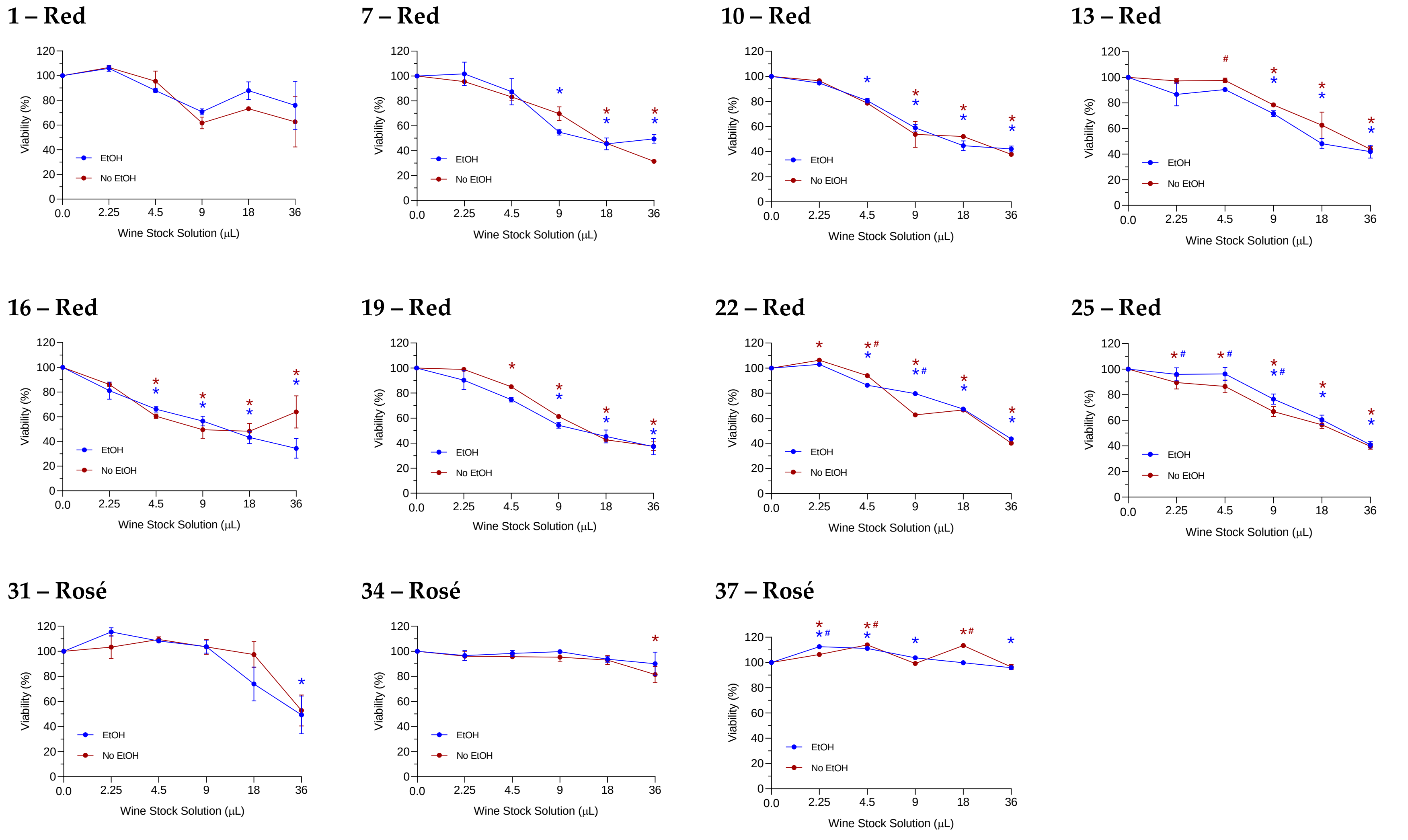

The results are summarised as three independent experiments' mean (± SEM). Results are reported as variation with respect to the untreated control (taken as 100 %). Statistical analysis: one-way ANOVA followed by Dunnett's post hoc for treated samples *vs*. control (untreated) cells (* $p < 0.05$, treated vs. control); paired sample *t*-test was used to determine the difference between EtOH-containing and EtOH-free extracts ($^{\#}$ $p < 0.05$, EtOH-containing *vs*. EtOH-free).

Supplementary Figure S4. Primary DNA damage in HepG2 cells after treatment with Wine Stock Solution.

1 – Red

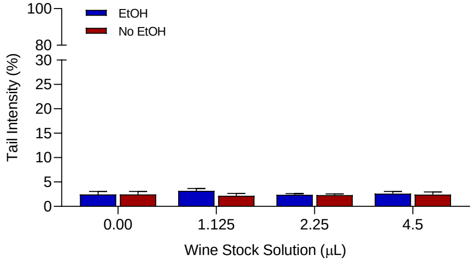

7 – Red

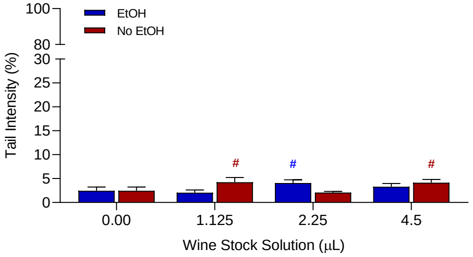

10 – Red

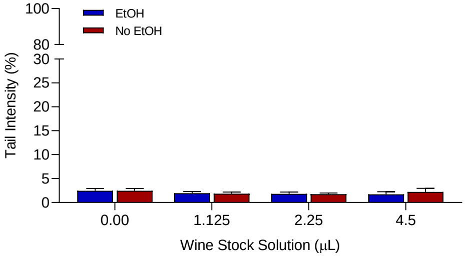

13 – Red

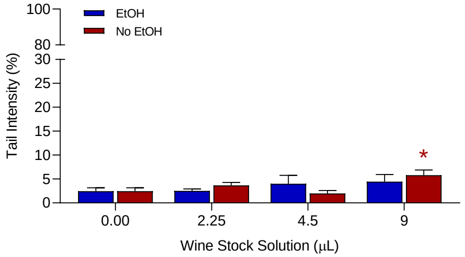

16 – Red

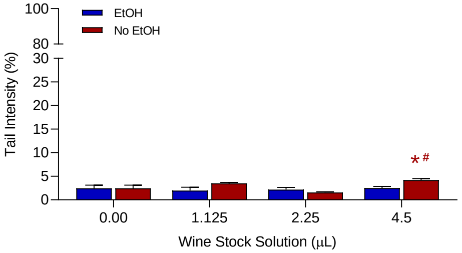

19 – Red

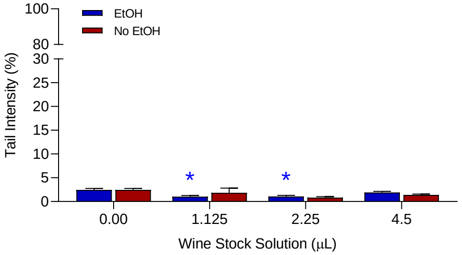

22 – Red

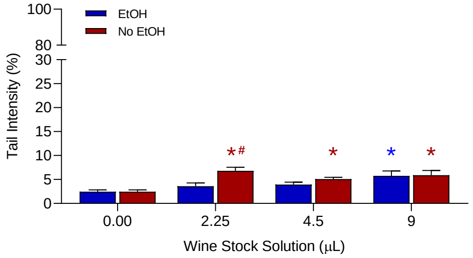

25 – Red

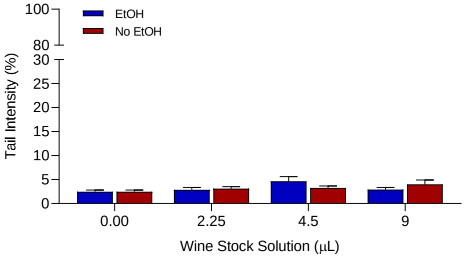

31 – Rosé

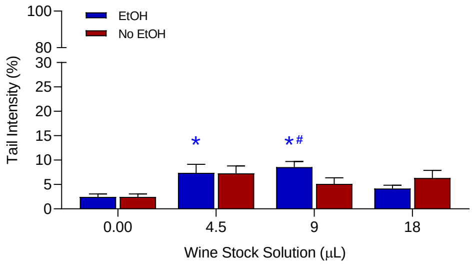

34 – Rosé

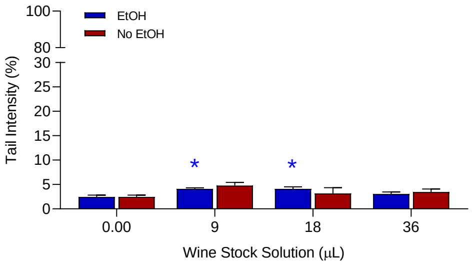

37 – Rosé

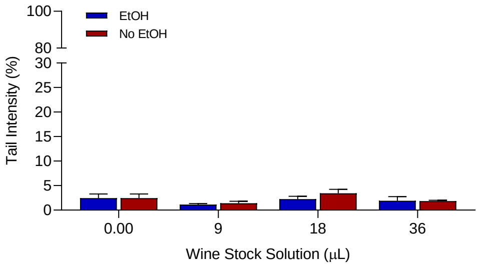

Tail intensity (% DNA) was used as indicator of primary DNA damage. The results are summarised as three independent experiments' mean (± SEM). Statistical analysis: one-way ANOVA followed by Dunnett's post hoc for treated samples *vs.* control (untreated) cells (* $p < 0.05$, treated vs. control); paired sample *t*-test was used to determine the difference between EtOH-containing and EtOH-free extracts (# $p < 0.05$, EtOH-containing *vs.* EtOH-free). Tail intensity of positive control (1 μM 4NQO): 12.31 ± 1.15 ($p < 0.001$ *vs.* untreated cells, *t*-test)
